# Prolonged corticosteroid treatment in acute respiratory distress syndrome: impact on mortality and ventilator-free days

**DOI:** 10.1186/s13054-018-2007-z

**Published:** 2018-05-24

**Authors:** Gianfranco Umberto Meduri, Bram Rochwerg, Djillali Annane, Stephen M. Pastores, on behalf of the Corticosteroid Guideline Task Force of SCCM and ESICM

**Affiliations:** 1 Division of Pulmonary, Critical Care, and Sleep Medicine, Department of Medicine, Memphis Veterans Affairs Medical Center, (111) - 1030 Jefferson Avenue Suite room #CW444 -, Memphis, TN 38104 USA; 2 Division of Critical Care, Department of Medicine, McMaster University, Hamilton, ON Canada; 3 General ICU Department, Raymond Poincaré hospital (APHP), Health Science Centre Simone Veil, Université Versailles SQY-Paris Saclay, Paris, France; 4 Department of Anesthesiology and Critical Care Medicine, Memorial Sloan Kettering Cancer Center, New York, NY USA

We read with interest the letter by Blot and colleagues recently published in *Critical Care* [1] which concluded that the recommendations of the Corticosteroid Guideline Task Force of SCCM and ESICM [2] for the use of adjunctive corticosteroids in early moderate to severe acute respiratory distress syndrome (ARDS) were based on insufficient evidence. In support of their view, the authors refer to the meta-analysis of Ruan et al. (reference [3] in Blot and colleagues’ letter) also published in *Critical Care* in 2014. We respectfully disagree with their comments and offer the following observations. First, the meta-analysis by Ruan et al. did not take into account how current understanding of disease pathophysiology impacts the administration of corticosteroid treatment in ARDS. The meta-analysis by Ruan et al. incorporated four randomized trials from the 1980s that investigated short-term (24–48 h) massive daily corticosteroid doses (up to 120 mg/kg methylprednisolone equivalent), an intervention that is obsolete and discredited by the present pathophysiological understanding of ARDS [2]. Thus, the inclusion of these trials in the meta-analysis is mostly responsible for the inconsistency reported in their letter. Moreover, the conclusion by Ruan et al. that the benefits of corticosteroid treatment decreased over time are not supported by the actual findings of the cited trials (Figure 3 in [4], Figure 4 in [5] and Table 5 in [3]).

**Fig. 1:**
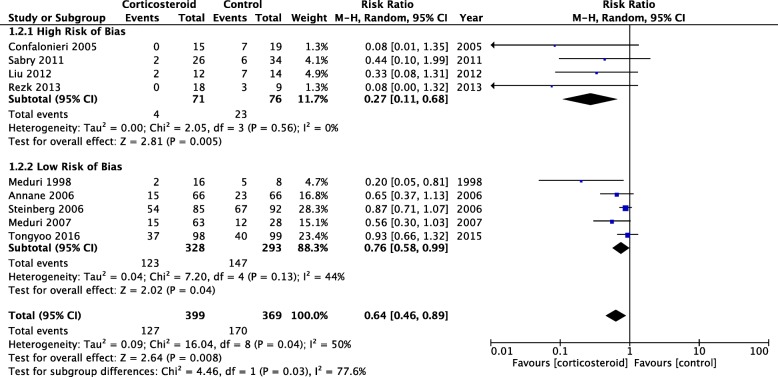
Hospital mortality in ARDS subgroup before day 14. Comparison between randomized trials at high risk of bias versus those at low risk of bias which investigated prolonged glucocorticoid (methylprednisolone or hydrocortisone) treatment in ARDS

As the Corticosteroid Guideline Task Force state in the guideline, our recommendation for adjunctive use of corticosteroids in early moderate to severe ARDS is a conditional recommendation and not necessarily meant to imply a standard of care treatment. In our analysis [2] the pooled relative risk estimate for hospital mortality (Fig. 1) with corticosteroids was 0.64 (95% confidence interval (CI) 0.46–0.89). Even if one excludes the four studies which Blot and colleagues appear to question [1], the pooled relative risk estimate for hospital mortality is 0.76 (95% CI 0.58–0.99), which is not significantly different from 0.64 (0.46–0.89). Independent of hospital mortality, the use of corticosteroids was associated with approximately a 7-day increase in ventilator-free days (mean difference 7.06 days, 95% CI 3.19–10.93) [2] (supplementary digital content in [5]). Given that our recommendation for adjunctive corticosteroids in ARDS is conditional in nature, ongoing and future prospective trials will certainly impact our future recommendations.
